# The Semantic Variant of Primary Progressive Aphasia: Clinical and Neuroimaging Evidence in Single Subjects

**DOI:** 10.1371/journal.pone.0120197

**Published:** 2015-03-10

**Authors:** Leonardo Iaccarino, Chiara Crespi, Pasquale Anthony Della Rosa, Eleonora Catricalà, Lucia Guidi, Alessandra Marcone, Fabrizio Tagliavini, Giuseppe Magnani, Stefano F. Cappa, Daniela Perani

**Affiliations:** 1 Vita-Salute San Raffaele University and Division of Neuroscience, San Raffaele Scientific Institute, Milan, Italy; 2 CERMAC, Vita-Salute San Raffaele University, Milan, Italy; 3 Istituto di Bioimmagini e Fisiologia Molecolare C.N.R., Segrate, Italy; 4 Istituto Universitario degli Studi Superiori—IUSS, Pavia, Italy; 5 Department of Clinical Neurosciences, San Raffaele Hospital, Milan, Italy; 6 IRCCS Foundation “Carlo Besta” Neurological Institute, Milan, Italy; 7 Departments of Neurology, San Raffaele Hospital, Milan, Italy; 8 Nuclear Medicine Unit, San Raffaele Hospital, Milan, Italy; University G. D'Annunzio, ITALY

## Abstract

**Background/Aim:**

We present a clinical-neuroimaging study in a series of patients with a clinical diagnosis of semantic variant of primary progressive aphasia (svPPA), with the aim to provide clinical-functional correlations of the cognitive and behavioral manifestations at the single-subject level.

**Methods:**

We performed neuropsychological investigations, ^18^F-FDG-PET single-subject and group analysis, with an optimized SPM voxel-based approach, and correlation analyses. A measurement of white matter integrity by means of diffusion tensor imaging (DTI) was also available for a subgroup of patients.

**Results:**

Cognitive assessment confirmed the presence of typical semantic memory deficits in all patients, with a relative sparing of executive, attentional, visuo-constructional, and episodic memory domains. ^18^F-FDG-PET showed a consistent pattern of cerebral hypometabolism across all patients, which correlated with performance in semantic memory tasks. In addition, a majority of patients also presented with behavioral disturbances associated with metabolic dysfunction in limbic structures. In a subgroup of cases the DTI analysis showed FA abnormalities in the inferior longitudinal and uncinate fasciculi.

**Discussion:**

Each svPPA individual had functional derangement involving an extended, connected system within the left temporal lobe, a crucial part of the verbal semantic network, as well as an involvement of limbic structures. The latter was associated with behavioral manifestations and extended beyond the area of atrophy shown by CT scan.

**Conclusion:**

Single-subject ^18^F-FDG-PET analysis can account for both cognitive and behavioral alterations in svPPA. This provides useful support to the clinical diagnosis.

## Introduction

In the mid-1970s, Endel Tulving proposed the concept of semantic memory [1]. After this insightful work, Elizabeth Warrington reported three patients in which “*a selective impairment”* of this cognitive function was the prominent clinical finding [2]. A detailed cognitive evaluation of the syndrome of semantic dementia was first provided by Snowden and co-workers in 1989 [3]. Three years later, Hodges et al. described several cases with similar semantic disruption showing circumscribed atrophy of temporal poles (TPs) in 1992 [4]. The syndrome was recognized as one of the clinical presentations of frontotemporal dementia [5] and later classified as the semantic variant of primary progressive aphasia by Gorno-Tempini et al. [6]. From a clinical standpoint, patients suffering from svPPA with prevalent involvement of the left hemisphere (left svPPA) usually present with severe anomia, word-finding difficulties, and impaired single word comprehension (so-called *loss of memory for words*) [7–9]. In the case of the right hemispheric variant (right svPPA), the patients typically present with defective recognition of familiar and famous faces [10,11]. This is often in addition to bizarre food choices, food restrictions, and eating preferences (rather than binge eating, which is typical of bvFTD) [12–14]. Abnormal interests in jigsaw puzzles and clockwatching have also been reported [15]. In addition, there is a growing body of research about the behavioral alterations often exhibited by svPPA patients [16–20]. Clinico-pathological studies have shown that FTLD TAR-DNA binding protein (FTLD-TDP) is the underlying pathology in about 68–80% of clinically diagnosed individuals with svPPA [21–23].

The neurodegeneration process of svPPA is known to progressively involve bilateral temporal lobes, as confirmed by a large amount of imaging studies, showing coherent patterns of structural/functional abnormalities in the brain involving the temporal poles (TPs) [16,24–33], hippocampal and parahippocampal structures [24,25,28,29,32] and the anterior inferior, middle and superior temporal gyri (aITG, aMTG and aSTG) [16,24–26,28,29,31,33,34]. As the disease progresses, the neurodegeneration may reach additional limbic regions, such as amygdala [16,25,28,29,31,33,34], the insular complex [16,28,29,31,32,34], the ventromedial prefrontal cortex (mainly orbitofrontal; vmPFC, OFC) [16,25,28,29,31,32,34] and the cingulate cortex [32]. Sometimes the basal ganglia (caudate nuclei [25,29]) are also involved. White matter alterations have been found to mirror temporal lobe grey matter abnormalities, mainly affecting inferior longitudinal and uncinate fasciculi integrity [24,35–37]. Recent works have also highlighted decreased functional connectivity in caudate nucleus and FFG [38,39].

Since the first landmark studies [40,41] ^18^F-FDG-PET functional investigations have provided consistent findings of bilateral hypometabolism in temporal lobes, peaking in the TPs, and usually prevalent to the left [24,42–48]. Some authors have reported an additional involvement of the basal ganglia (caudate nucleus), thalamus [42,43,45], subcallosal gyrus/orbitofrontal cortex (SCG, OFC) [24,42,44,46], insula [42,49], anterior cingulate cortex (aCG) [50], and fusiform gyrus (FFG) [24,43,44,46,47].

It is of note that these findings are mostly based on group-analysis or case reports and none of them resulted from optimized voxel-based procedures applied at the single-subject level. Despite the value of parametric group-based investigations, it would be useful to develop single-subject analysis routines for clinical purposes. Therefore, our goal here is to demonstrate the value of a single-subject ^18^F-FDG-PET assessment in detecting functional abnormalities of glucose metabolism and their relation with the cognitive and behavioral disturbances in each svPPA patient. This could prove to be useful in clinical practice for differential and early diagnosis, especially in the case of patients with atypical presentations.

The aims of this work are: 1) To contribute a study with ^18^F-FDG-PET imaging using an optimized statistical parametric mapping (SPM) procedure at the single-subject and group-level in a cohort of clinically diagnosed svPPA [6]; 2) To test the correlation of performance in semantic tasks with specific brain metabolic alterations; 3) To evaluate the relationship between ^18^F-FDG-PET metabolic patterns as shown by SPM-t maps and CT atrophy.

Additionally, in a subgroup of patients we could test the status of white matter in the uncinate and inferior longitudinal fasciculi (UF and ILF) and their relationship with ^18^F-FDG-PET metabolic patterns. This is relevant given the involvement of these pathways in svPPA neurodegeneration, cognitive and behavioral disturbances.

## Participants

The cohort included 10 patients (N = 4 males), mean age 67.00, standard deviation (SD) 9.13, age range 58–85, mean education 11.40 years; SD 3.92; range 5–17. They all fulfilled the criteria for svPPA [6]. The mean age at onset was 64.10; SD 10–07 (range 53–82). All the patients were evaluated at the San Raffaele Hospital (Milan, Italy) between April 2009 and July 2013 (^18^F-FDG-PET time) or at the IRCCS BESTA Foundation, Milan, Italy. One of the patients exhibited a novel missense progranulin gene mutation, which was described in a previous work [51]. All patients underwent a neurological examination, ^18^F-FDG PET/CT scan, and a detailed neuropsychological assessment. Three patients also underwent MRI Diffusion Tensor Imaging acquisition. All patients were right-handed. For a demographic summary see Table 1.

**Table 1:** Demographic summary of the studied cohort.

Index	svPPA (9 L>R, 1 R>L)
**MMSE**	24,29±3,40 (18–28.27)
**Age (yrs)**	67.00±9.13 (56–85)
**Age at onset (yrs)**	64.10±10.07 (53–82)
**Disease Duration (yrs)**	2.95±1.42 (0.5–4)
**Years of Education**	11.40±3.92 (5–17)

Statistics are indicated as follows: Mean ±SD (RANGE)

L>R: Left Hemisphere hypometabolism Asymmetry

R>L: Right Hemisphere hypometabolism Asymmetry

## Methods

### Cognitive Assessment

The cognitive evaluation was based on a detailed neuropsychological battery. This included tests for language (Token Test) [52,53] reasoning (Raven Colored Progressive Matrices [54], letter and category fluency test, [55]), short-term verbal memory (Digit Span Forward, [56] or Rey-Auditory Verbal Learning Test, RAVLT immediate [57], visuo-spatial short-term memory (Corsi Test [56]), verbal long-term memory (RAVLT, delayed recall [57]), long-term visual spatial memory (Rey-Osterrieth Complex Figure recall [58]), visuoconstructive abilities (Rey-Osterrieth Complex Figure copy [58]), and attention (Attentive Matrices, [53]). Furthermore, patients underwent a specific test addressing semantic processing, namely the Pyramids and Palm Trees Test [59], a widely used tool for non-verbal semantic knowledge [60]. Patients underwent also a language test developed in our center which investigates semantic memory through Confrontation Naming and Comprehension tasks [61]. As quantitative neuropsychiatric assessments were not available, this aspect was assessed on the basis of clinical records information.

### ^18^F-FDG-PET imaging

#### Acquisition

All subjects underwent an ^18^F-FDG-PET imaging session, using 3D PET scans, either a General Electric Discovery LS PET/CT or a multi-ring General Electric Discovery STE PET/CT at the Department of Nuclear Medicine, San Raffaele Hospital, Milan, Italy. Patients received an intravenous injection of approximately 270 MBq of ^18^F-FDG (mean dose 250,60 MBq; SD: 56,41 range 179–351) in rest condition, lying supine in a quiet, dimly-lit room. Image acquisition started approximately 45min after injection, with a scan duration of 15 minutes. In particular, before radiopharmaceutical injection of ^18^F-FDG, subjects were fasted for at least 6 hours and measured blood glucose level threshold of <120 mg/dL. Image reconstruction followed an ordered subset expectation maximization (OSEM) algorithm. CT was co-registered and used for attenuation correction. Scatter correction was applied with software integrated in our scanner. The protocol has been approved by the San Raffaele Hospital Local Ethical Committee. All the patients gave informed written consent.

#### Single-subject analysis

Image analysis was carried out with SPM5 software (Wellcome Department of Imaging Neuroscience, London, UK; www.fil.ion.ucl.ac.uk/spm) on MATLAB 8 (MathWorks Inc, Sherborn, Mass). At the single subject level, ^18^F-FDG-PET imaging analysis has been performed according to an optimized SPM FDG-PET pipeline previously developed and validated [62]. In a first step, scans were ‘spatially normalized’ in accordance to a reference FDG-PET “dementia- specific” template [63].

In a second step, spatially normalized and smoothed images for a single patient were then compared to a large group of control scans by means of a two-sample t-test implemented in SPM5 to assess areas of hypometabolism throughout the whole-brain at a single-subject level. Proportional scaling was used to remove intersubject global variation in PET intensities. The threshold for assessing hypometabolism was set at p = 0.05, FWE-corrected for multiple comparisons at the voxel level. Only clusters containing more than 100 voxels were deemed to be significant. The resulting single-subject SPM hypometabolic maps have been also visually inspected by a team of experts (neurologists, radiologists, and nuclear medicine physicians) in PET imaging in order to further validate svPPA pathologically hypometabolic areas in each patient.

#### Group and commonalities analysis

We also assessed hypometabolism at the group-level evaluating: (1) group differences by means of a two-sample t-test between the group of svPPA patients vs. a subset of age-matched control subjects (i.e. N = 50: 5 HC/1 subject). A Family Wise Error threshold of P_FWE_<0.05 was applied in order to correct for multiple comparisons (cluster extent = 100 voxels) and (2) common areas of hypometabolism in the svPPA group using the contrast images resulting from each first-order single-subject assessment. For the latter, we used a one-sample t-test, setting age as a nuisance variable. The p-value (uncorrected) was lowered to p<0.001 and the minimum cluster size was of 100 voxels, given the small number of patients.

#### ROI-based correlation analysis

Correlation analysis was carried out with MarsBar toolbox [64] for SPM5 software, in a one-sample t-test design, using the single-subject hypometabolism contrast images (2^ND^ level analysis) and a covariate with confrontation naming test performances. We hypothesized the presence of a negative correlation, meaning that a greater level of decreased metabolism would correlate with a highly impaired performance. As one patient (Case 5) was tested with a naming task from a different battery (BADA, [65]), we used as covariate a vector containing a ratio between number of correct answers and maximum scores of the adopted tests. The ROIs analysis was run by selecting *a priori* a group of ROIs known to be associated with naming tasks in svPPA, together with regions commonly found hypometabolic in this condition. More specifically, we included the left temporal lobe (subdivided into ITG, MTG and STG), the FFG, IPL, caudate, amygdala and thalamus. Structural ROIs were obtained with the Wake Forest University PickAtlas (WFUPickAtlas) toolbox [66], using the Automated Anatomical Labeling (AAL) template [67] for SPM. Only individual clusters with a significance of P_uncorrected_<0.05 were deemed as significant.

### CT-PET imaging

Since a CT/PET scan was performed (see descriptions in PET imaging methods section) for attenuation correction, we evaluated the CT images for presence of atrophy. An experienced board certified neuroradiologist (DP) blind-rated the atrophy levels in the CT scans (4 atrophy levels in different brain regions: none, low, mild/moderate, high/severe).

### MRI Diffusion Tensor Imaging

#### Acquisition

A subgroup of patients (N = 3) and N = 20 age-matched healthy controls underwent a Diffusion Tensor Imaging (DTI) scan, which was performed with a 3-T Philips Achieva scanner (Philips Medical Systems, Best, NL) with an 8-channel head coil. Whole-brain DTI data was collected using a single-shot echo planar sequence (TR/TE = 8986/80 msec; FOV = 240 mm^2^; 56 sections; 2.5 mm isotropic resolution) with parallel imaging (SENSE factor, R = 2.5) and diffusion gradients applied along 32 non-collinear directions (b-value = 1000 sec/mm^2^). One non-diffusion weighted volume was also acquired.

#### Preprocessing and probabilistic tractography

Preprocessing and analysis of DTI data were performed via the FMRIB Software Library (FSL: http://fsl.fmrib.ox.ac.uk/fsl/fslwiki/‎) tools. Single-subject datasets were first corrected for eddy current distortions and motion artifacts, applying a full affine (linear) alignment of each volume to the no-diffusion weighting image.

We had *a priori* hypothesized the involvement of the inferior longitudinal fasciculus (ILF) and uncinate fasciculus (UF) in patients with svPPA, given the clinical features (naming difficulties and neuropsychiatric manifestations) and the abnormalities observed in ^18^F-FDG-PET result maps. Thus, we performed probabilistic tractography of the bilateral UF and ILF on 3 svPPA patients and 20 healthy controls, in order to test for possible fractional anisotropy (FA) and mean diffusivity (MD) changes in the fiber tracts of interest.

We used bedpostX/probtrackX to perform the multi fiber probabilistic tractography approach as described by Behrens et al. (2003, 2007) [68,69]. Both seed and termination cortical masks used to reconstruct the ILF (occipital and temporal poles) and the UF (temporal and frontal poles) were derived from the Harvard-Oxford Cortical and Subcortical Structural Atlas (http://fsl.fmrib.ox.ac.uk/fsl/fslwiki/Atlases).

Generated pathways are volumes in which values at each voxel represent the number of samples passing through that voxel and, therefore, the probability of connection to the seed voxel.

To remove spurious connections, the pathways in individual subjects were thresholded at 1% of the total number of generated tracts. The resulting thresholded tracts were then visually inspected to confirm that the pathways appeared anatomically correct, with no voxels outside of the expected pathway. Pathways in each subject were then binarized and averaged to produce a population probability map for each pathway. Voxel values in these maps mirror the proportion of subjects in whom a pathway is present. Group pathways were thresholded to include voxels present in at least 10% of participants and binarized to define a mask of each tract of interest.

In order to compare microstructural integrity between each single patient and the control group, we extracted mean values of FA and MD from each pathway and each subject. In particular, we employed *fslmeants* to mask the FA and MD whole-brain images with the probability maps of the ILF and UF. This allowed us to obtain mean values in all subjects for each pathway and measure. Finally, single patients’ values were compared to either the 5^th^ percentile (FA index) or the 95^th^ percentile (MD index) of the controls’ values distribution.

### Results

#### Cognitive Assessment

Deficits in confrontation naming and/or categorical verbal fluency were present in all patients. This was associated with a relative sparing of phonemic fluency scores, a feature which has been considered as typical of svPPA, particularly in the early stages of the progression [70]. The non-pathological MMSE mean score (24.29; SD = ±3.40) is consistent with the literature [30,71]. Six out of ten patients underwent the PPT semantic memory test and four presented with a pathological score. To summarize, a prominent disorder of semantic memory, as shown by defective performance in tests like categorical fluency, naming or word-picture matching, was found in all the patients. With a few exceptions (3/10, Case 1,4 and 8), we found a relative sparing of the other cognitive domains (e.g. attention or visuo-constructive abilities). RAVLT testing data was available for 6/10 patients. Pathological performance at immediate recall subscore was shown by 3/6 patients, whereas 5/6 had impaired delayed recall subscores. Case 10 reported impaired recognition of her relatives on several occasions, therefore showing signs of prosopagnosia (suspected right variant svPPA). Overall the pattern of cognitive alteration was very consistent and is shown in Table 2.

**Table 2:** Neuropsychological testing results from the 10 cases presented.

	Cut-off Score	Case 1	Case 2	Case 3	Case 4	Case 5	Case 6	Case 7	Case 8	Case 9	Case 10
**MMSE**	**>24**	29*	27.53	24.49	28,27	**18**	**23,53**	**21.20**	**20.86**	24.85	27*
**Token Test**	**>29.25**	**27.25**	34	31.25	**25,75**	**23**	29.75	**17.5**	**16.25**	34	31.25
**Digit Span Forward**	**>4.25**	6.5	4	5.75	6.29	**Path** °	5.23	5.75	**3.25**	6.99	4.87
**Corsi Test**	**>4.25**	**4**	5.25	**3.75**	-	Norm°	**3.25**	**4**	**3.25**	5.94	4.87
**RAVLT (immediate)**	**>28.53**	-	**21.3**	**26.4**	**20.4**	-	-	-	30.9	30.3	42.1
**RAVLT (recall)**	**>4.69**	-	**1.7**	**2**	**2.2**	-	-	-	**3.6**	**1.8**	9.3
**Naming (CAGI)**	**>43.99**	**14.21**	**31.21**	**12.21**	**12.287**	**Path§**	**36.41**	**8**	**17.82**	**35.05**	**37.23**
**Comprehension (CAGI)**	**>47.39**	**41.927**	48	**44.979**	**43.083**	**Path§**	48	**36**	**44.018**	48	**46.018**
**Phonemic Fluency**	**>23**	**17**	34	27	**21**	**0**	**22**	**0**	26	27	**13** *
**Semantic Fluency**	**>30**	**11**	**18**	**8**	31	**9**	**19**	**0**	**11**	**23**	**16** *
**Palm and Pyramids Test**	**>40.15**	22**	**37**	26**	**37.47**	-	-	**16** **	-	-	**27.66**
	**>18** **										
**Rey Figure (Copy)**	**>30.05**	**28.5**	34	31.25	**21.5**	Norm°	30.5	37.5	34.25	34.54	36
**Rey Figure (Recall)**	**>11.23**	16.25	12	14.75	12.25	-	12.75	17.5	19.5	**0**	19
**Raven Matrices**	**>23.25**	34	37	22.5	34	24.3	27.5	30.5	29.5	-	21*
**Attentive Matrices**	**>37**	46	46.25	49	41.25	49.4	41.75	41.25	**28.50**	37.75	40.75

All tests scores are corrected referring to respective Italian normative data (see methods).

*: These are raw scores, as it was not possible to correct scores for Years of Education and Age.

**: These scores refer to a modified version of the PPT.

§: This patient underwent similar naming and comprehension tests from a different battery (BADA); see text for details.

°: Numeric scores were not available.

**Bold scores** are pathological.

### ^18^F-FDG PET imaging

#### Single-subject analysis

**Fig 1:**
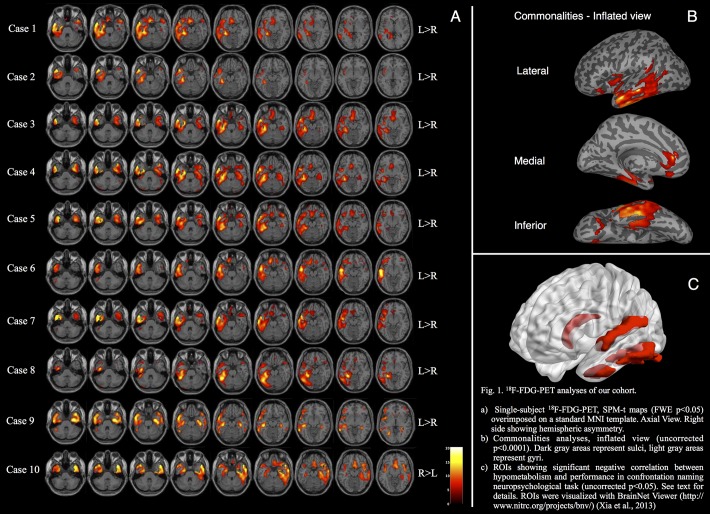
^18^F-FDG-PET analyses of our cohort. **A)** Single-subject ^18^F-FDG-PET, SPM-t maps (FWE p<0.05) overimposed on a standard MNI template. Axial View. Right side showing hemispheric asymmetry. **B)** Commonalities analyses, inflated view (uncorrected p<0.0001). Dark gray areas represent sulci, light gray areas represent gyri. **C)** ROIs showing significant negative correlation between hypometabolism and performance in confrontation naming neuropsychological task (uncorrected p<0.05). See text for details. ROIs were visualized with BrainNet Viewer (http://www.nitrc.org/projects/bnv/) [104].

Each patient showed unilateral or bilateral involvement of temporal poles (with varying degrees of asymmetry, see Fig. 1A), with additional extension to the left lateral temporal (inferior, middle and superior temporal gyrus and inferior fusiform gyrus) and medial temporal lobe regions (hippocampal formations). More specifically, in the left hemisphere, we found hypometabolism in the subiculum (7/10 patients), entorhinal cortex (5/10), and hippocampus proper (CA, 4/10). In the right hemisphere, we found metabolic decreases in fewer cases, namely 3/10 patients in the subiculum, 2/10 at entorhinal cortex and 4/10 in CA. Single-subject SPM t-maps evaluation revealed additional regional hypometabolism in limbic structures (i.e. insula, amygdala, ACG or OFC) of 8/10 patients and in IPL and temporo-parietal junction (TPJ) of 6/10. Case 10 showed an asymmetrical hypometabolism pattern prevalent in the right temporal lobe, consistent with her clinical presentation.

#### Group analysis

*(1)* Group-differences confirmed the bilateral involvement of the TPs (TP cluster significant at P_FWE_<0.05) and an extended hypometabolism in the left temporal lobe (inferior, medial and antero-supero-lateral aspects). This was together with a significant involvement of the OFC (see Table 3 for details) for the svPPA subjects with respect to the age-matched group of controls. *(2)* In the svPPA group, we found a common extensive hypometabolic cluster in the left hemisphere. This involved the inferior and middle temporal regions, insula, anterior cingulate regions. In addition, the OFC was significantly hypometabolic (p<0.001 uncorrected, see Fig. 1B and Table 3 for details).

**Table 3:** ^18^F-FDG-PET group and commonalities SPM analyses.

Analysis	P-value	MNI coordinates	T	K	Label
		(x,y,z)			
**(1) Group**	**P**_**FWE**_ **<0.05**	−52,8,−26	12.39	4677	Temporal_Mid_L
					Fusiform_L
					Temporal_Inf_L
					Temporal_Pole_L
					Parahippocampal_L
		52,18,−22	8.56	544	Temporal_Pole_Sup_R
					Fusiform_R
					Temporal_Inf_R
		0,24,−18	7.25	439	Rectus_L
**(2) Commonalities**	**P**_**UNCORR**_ **<0.001**	−8,40,10	19.43	7855	Cingulum_Ant_L
					Temporal_Mid_L
					Rolandic_Operculum_L
					Temporal_Inf_L
					Temporal_Pole _L

Location, T-value and cluster size (K) of peaks of significant hypometabolism in svPPA group, divided by means of analysis approach. Labels are obtained by WFUPickAtlas SPM5 Toolbox. See Methods in text for details.

#### ROI-based analysis

Correlation analyses showed significant negative correlation between naming task scores and hypometabolism in the left ITG (p-value<0.045), STG (p-value<0.009), FFG (p-value<0.017) and caudate nucleus (p-value<0.009) (see Table 4 and Fig. 1C).

**Table 4:** Table showing ROIs significantly correlated to Confrontation Naming Test percentages scores.

Hypothesis	Analysis	Region	P-value	x	y	z
**Negative correlation between Hypometabolism and Confrontation Naming Tests**	**ROI-based**	L_ITG	<0.045	−50	−29	−25
		L_STG	<0.009	−53	−22	6
		L_FFG	<0.017	−40	−48	−22
		L_CAU	<0.009	−12	10	8

Significance was set at P uncorrected <0.05. Structural Regions of Interest were extracted by normalized and published ATLAS (see text for details). P-values and coordinates are relative to the activation peaks of each ROI (see also Fig. 1C).

### Structural imaging

#### CT ratings

Atrophy ratings confirmed a moderate to severe atrophy in the left TP, left anterior STG, and in left insula in more than 50% of the sample. No atrophy was reported for FFG and OFC. Mild atrophy was reported in the left frontal operculum for 30% of the patients. Left medial temporal regions (hippocampal and parahippocampal structures) were atrophic in all the patients (60% mild atrophy, 40% moderate atrophy). No atrophy was evident in the right hemisphere.

#### MRI DTI imaging

Probabilistic tractography revealed a lower MD index in the ILF in all 3 cases, while in the UF in 2/3 patients. FA index showed abnormal values in both UF and ILF in all the subjects (see Table 5 for details).

**Table 5:** Probabilistic Tractography Results.

**ProbabilisticTractography**	**A – FA mean values**	**HC (mean)**	**HC (St.Dev)**	**HC (5** ^**th**^ **Percentile)**	**Case 1**	**Case 3**	**Case 10**
	Right UF_FA	0.354044	0.022527	0.313960	0.321478	0.368426	0.268635*
	Left UF_FA	0.355275	0.022342	0.323846	0.318266*	0.34695	0.281353*
	Right ILF_FA	0.401055	0.019568	0.369104	0.366063*	0.368585*	0.323986*
	Left ILF_FA	0.409574	0.020515	0.373823	0.38536	0.372257*	0.328735*
	**B – MD mean values**	**HC (mean)**	**HC (St.Dev)**	**HC (95** ^**th**^ **Percentile)**	**Case 1**	**Case 3**	**Case 10**
	Right UF_MD	0.000880	0.000048	0.000972	0.000999592*	0.000917813	0.00118558*
	Left UF_MD	0.000862	0.000041	0.000939	0.00107492*	0.00104544*	0.00103267*
	Right ILF_MD	0.000851	0.000033	0.000909	0.000945382*	0.000879776	0.00112745*
	Left ILF_MD	0.000839	0.000031	0.000891	0.00101494*	0.000986195*	0.000982657*

A) mean FA values and B) mean MD values extracted from the bilateral uncinate fasciculus and the bilateral inferior longitudinal fasciculus. From left to right, columns indicate mean values in control group, standard deviation in control group, 5^th^ percentile (FA: lower FA, lower white-matter integrity) or 95^th^ percentile (MD values: higher the MD index, greater the microstructural degeneration) of the distribution of microstructural indices in healthy controls, values of case 1, values of case 3, values of case 10. Patients’ values being lower than the 5^th^ percentile (FA) or higher the 95^th^ percentile (MD) of the distribution healthy controls’ values are marked in red.

* marks significant values.

## Discussion

Our svPPA cohort showed a prominent deterioration of semantic memory, which is the typical sign of this neurodegenerative syndrome. The preservation of executive-attentive abilities shown by svPPA patients suggests that semantic fluency difficulties may be accounted for by loss of semantic vocabulary rather than by executive deficits [71]. Verbal and spatial short-term memory domains were generally spared in our cohort (only 2/10 patients performed pathologically). The core criteria for a diagnosis of svPPA are the naming difficulties associated with deficits in single-word comprehension [6]. The confrontation naming test [61] was the most efficient tool in revealing the core semantic difficulties of svPPA patients. Single-word comprehension deficits were shown in 7 out of 10 patients. In the early stages of the disease, defective comprehension may be present only for low frequency words, leading to an underdiagnosis of the condition [22,72].

The group analysis of ^18^F-FDG PET yielded results consistent with the available literature [24,42–44,47], showing selective hypometabolism in the anterior temporal lobes (TPs bilaterally), medial temporal lobes, the lateral inferior temporal cortex, extending to the temporo-occipital junction, and the limbic structures (insula, OFC and anterior cingulate region) (see Table 2 and Fig. 1B).

The CT ratings confirmed a significant loss of gray matter in ATL regions, particularly in the temporal pole (on the left side) and at the medial temporal aspects level. No atrophy was revealed in FFG, confirming previous evidence (see, among the others, [24,32]). The partial discordance between the metabolic derangement and the gray matter loss may be accounted for by neural disconnection induced by early pathological events occurring in svPPA, particularly in the ATLs. The integrity of the connections seeding from these areas and projecting to frontal and posterior associative areas (through UF and ILF, respectively) appears to be altered, as shown in the patients who underwent MRI-DTI. This system disconnection and the consequent neuronal dysfunctions seem to be at the basis of the cognitive and behavioral alterations exhibited by the patients.

Consistent with the naming and comprehension deficits, all the patients showed, at a single-subject level, a functional derangement encompassing the left temporal lobe, particularly in its anterior (temporal pole, superior temporal gyrus) and lateral inferior components (ITG and FFG). We further tested for associations between naming performances and hypometabolism, observing that the glucose metabolism in left ITG, STG, FFG and caudate nucleus correlated with confrontation naming scores. Significant correlations between naming scores and temporal lateral hypometabolism in svPPA have also been highlighted in previous works [28,46]. The role for the infero- and superior-lateral aspects of ATL, namely the ITG and STG, as a crucial hub in language lexical retrieval has been recently suggested by Mesulam [22]. The author claims that svPPA atrophy “challenges current concepts related to the neurology of language” and, therefore, that the left ATL should be considered as a third hub with a critical role within the language network [22]. Moreover, ITG and FFG are parts of the ‘basal temporal language area’ [73], which has a critical role in object naming and semantic processing. This region is part of the ventral stream, as shown by functional and structural imaging in both svPPA cohorts [28,32,46,74] and healthy controls [75–79]. Additionally, our analysis revealed a role for the left caudate. Some studies have already provided evidence for gray matter loss at caudate level in svPPA patients [25,29]. The negative correlation between caudate/FFG hypometabolism and naming performance might reflect a control role of the left caudate-left FFG circuit [80]. There is multiple evidence for a role of this circuit in suppressing words’ production in reading tasks in bilingual settings [81,82]. Several other studies have provided evidence for a role of the caudate nucleus in several language tasks, from phonological processing [83] to word generation [84]. Case 10 is of particular interest, as this patient showed a right hemispheric dysfunction, loss of memory for words, and a remarkable prosopagnosia (she was not able on several occasions to recognize her relatives). This cognitive alteration in early stages of the disease is typical of the right variant svPPA, and our single-subject ^18^F-FDG-PET analysis showed a predominant metabolic dysfunction in the right temporal pole, superior temporal gyrus and of the OFC (see Fig. 1A). The OFC cortex involvement was also coherent with irritability and mood deflections reported in the clinical history.

The single-subject ^18^F-FDG PET analyses also revealed a consistent hypometabolism of the medial temporal lobes, precisely in the entorhinal cortices, hippocampal subiculum or CA (see Results for details). Coherently, some of our patients showed a defective performance in long-term verbal memory (RAVLT delayed recall), a finding associated with damage to specific regions of the hippocampal formation [85,86]. The defective performance on episodic verbal memory task can, at least, in part, be accounted for by the language impairment. The notion of relative preservation of episodic memory in svPPA, and of the corresponding neural correlates, is currently the focus of intensive investigation [87–89].

It is noteworthy that 80% of svPPA cases showed hypometabolism in limbic regions such as the amygdala, insula or OFC. All these patients presented with a history of neuropsychiatric manifestations, such as anxiety, depression or mood disturbances. A limit of this study, however, is that these behavioral disturbances were not quantified. Still, we observed that reported anxiety, agitation or mood disorders by the single patient were always coupled with regional hypometabolism at the single subject level in limbic areas. The behavioral alterations have been initially underestimated in svPPA patients, given the focus on the isolated language difficulties. These abnormalities have been subsequently highlighted [20], and it is now recognized that svPPA progression usually leads to behavioral manifestations [22]. Further studies are needed to investigate these behavioral disturbances and the neurofunctional correlates in svPPA.

Noteworthy, the functional involvement of limbic structures here shown in most svPPA individuals by ^18^F-FDG PET suggests that the neurodegenerative process extends beyond the anterior aspects of the temporal pole. This area is heavily interconnected with other limbic regions and it is known to be a cortical convergence zone [90,91]. The temporal poles, initially considered a single area (BA 38), have recently been parceled out into several subregions on the basis of cytoarchitectonic differences [92] and connectivity-based approaches [93]. A recent resting-state fMRI study [94] evaluated the large-scale networks involving these subregions. At least four major temporal pole subregions, namely dorsal (auditory/somatosensory and language network connections), ventromedial (mainly visual networks), medial (paralimbic structures) and anterolateral (connected to default-semantic network), were delineated by this approach. In the case of the medial and anterolateral subregions, the uncinate fasciculus plays a crucial connection role and our MRI-DTI data support this. The UF is considered to be a component of the perisylvian language network [95] and has been specifically related to semantic memory retrieval [96,97]. The UF is also involved in socio-emotional processing [98]. A reduction of white matter integrity in the UF has been shown in the frontotemporal dementia spectrum, including in patients with svPPA [24,37,99,100]. Noteworthy, it has been also associated to behavioral symptoms (i.e. inhibition) [101]. In addition, the involvement of ILF—i.e., a component of the semantic ventral stream—seems also to be a prominent feature of the svPPA [37]. The ILF, connecting occipital and temporal regions, is thought to link object representations with their lexical labels [95]. It is also engaged in visual perception, face recognition, and reading [102]. More importantly, these white matter abnormalities may well contribute to the clinical presentation, and may be related to ^18^F-FDG PET hypometabolism.

In conclusion, the neurodegeneration in the svPPA encompasses a widespread network, ranging from visual to limbic pathways, and affecting bilaterally the temporal lobes. Our findings indicate an extension of degeneration both caudally (towards TPJ and IPL), and rostrally, reaching first OFC and then fronto-medial cortex.

## Conclusions

Our single-subject clinico-functional analysis highlighted the distinctive features of svPPA syndrome. We showed that convergent system abnormalities, from altered connectivity to metabolic dysfunction, characterize this degeneration and are associated with specific cognitive deficits. The alterations in the white matter connections within limbic regions, particularly through the uncinate fasciculus, might be the basis of the frontal extension of the neurodegeneration observed in this syndrome. Convergent findings from clinical assessment, structural imaging, and functional imaging in single cases are of utmost importance for both research and clinical practice. In the case of research, the study of svPPA has enormously contributed to the understanding of the neural basis of semantic memory [103]. In clinical practice, the coherence of imaging findings with patterns of cognitive alterations at single-subject level has an important supportive role for correct diagnosis and patient management. The ^18^F-FDG PET metabolic patterns in svPPA can help in the differential diagnosis in early disease phases, improving the definition of the ‘diagnostic boundaries’ [30] in the FTD spectrum.
